# Low-Dose vs Standard-Dose Alteplase in Acute Lacunar Ischemic Stroke: The ENCHANTED Trial

**DOI:** 10.1212/WNL.0000000000011598

**Published:** 2021-03-16

**Authors:** Zien Zhou, Candice Delcourt, Chao Xia, Sohei Yoshimura, Cheryl Carcel, Takako Torii-Yoshimura, Shoujiang You, Alejandra Malavera, Xiaoying Chen, Maree L. Hackett, Mark Woodward, John Chalmers, Jianrong Xu, Thompson G. Robinson, Mark W. Parsons, Andrew M. Demchuk, Richard I. Lindley, Grant Mair, Joanna M. Wardlaw, Craig S. Anderson

**Affiliations:** From The George Institute for Global Health, Faculty of Medicine (Z.Z., C.D., C.X., S. Yoshimura, C.C., T.T.-Y., A.M., X.C., M.L.H., M.W., J.C., C.S.A.), and South Western Clinical School (M.W.P.), University of New South Wales Sydney, Australia; Department of Radiology (Z.Z., J.X.), Ren Ji Hospital, School of Medicine, Shanghai Jiao Tong University, China; Department of Neurology (C.D., C.C., C.S.A.), Royal Prince Alfred Hospital, Sydney Health Partners; Sydney Medical School (C.D., C.C.), University of Sydney, Australia; Department of Neurosurgery (C.X.), West China Hospital, Sichuan University, Chengdu, China; Department of Cerebrovascular Medicine (S. Yoshimura, T.T.-Y.), National Cerebral and Cardiovascular Center, Osaka; Department of Neurology and Neuroscience (T.T.-Y.), Nagoya City University Graduate School of Medical Science, Japan; Department of Neurology (S. You), the Second Affiliated Hospital of Soochow University, Suzhou, China; The George Institute for Global Health, School of Public Health (M.W.), Imperial College, London; Department of Cardiovascular Sciences and NIHR Leicester Biomedical Research Center (T.G.R.), University of Leicester, UK; Melbourne Brain Centre, Royal Melbourne Hospital University Department of Medicine (M.W.P.), University of Melbourne, Australia; Departments of Clinical Neurosciences and Radiology, Hotchkiss Brain Institute, Cumming School of Medicine (A.M.D.), University of Calgary, Canada; Westmead Applied Research Centre (R.I.L.), University of Sydney, Australia; Division of Neuroimaging Sciences, Edinburgh Imaging and Centre for Clinical Brain Sciences (G.M., J.M.W.), and UK Dementia Research Institute (J.M.W.), University of Edinburgh; and The George Institute China at Peking University Health Science Center (C.S.A.), Beijing, China.

## Abstract

**Objective:**

To determine any differential efficacy and safety of low- vs standard-dose IV alteplase for lacunar vs nonlacunar acute ischemic stroke (AIS), we performed post hoc analyzes from the Enhanced Control of Hypertension and Thrombolysis Stroke Study (ENCHANTED) alteplase dose arm.

**Methods:**

In a cohort of 3,297 ENCHANTED participants, we identified those with lacunar or nonlacunar AIS with different levels of confidence (definite/according to prespecified definitions based on clinical and adjudicated imaging findings. Logistic regression models were used to determine associations of lacunar AIS with 90-day outcomes (primary, modified Rankin Scale [mRS] scores 2–6; secondary, other mRS scores, intracerebral hemorrhage [ICH], and early neurologic deterioration or death) and treatment effects of low- vs standard-dose alteplase across lacunar and nonlacunar AIS with adjustment for baseline covariables.

**Results:**

Of 2,588 participants with available imaging and clinical data, we classified cases as definite/probable lacunar (n = 490) or nonlacunar AIS (n = 2,098) for primary analyses. Regardless of alteplase dose received, lacunar AIS participants had favorable functional (mRS 2–6, adjusted odds ratio [95% confidence interval] 0.60 [0.47–0.77]) and other clinical or safety outcomes compared to participants with nonlacunar AIS. Low-dose alteplase (versus standard) had no differential effect on functional outcomes (mRS 2–6, 1.04 [0.87–1.24]) but reduced the risk of symptomatic ICH in all included participants. There were no differential treatment effects of low- vs standard-dose alteplase on all outcomes across lacunar and nonlacunar AIS (all *p*_interaction_ ≥0.07).

**Conclusions:**

We found no evidence from the ENCHANTED trial that low-dose alteplase had any advantages over standard dose for definite/probable lacunar AIS.

**Classification of Evidence:**

This study provides Class II evidence that for patients with lacunar AIS, low-dose alteplase had no additional benefit or safety over standard-dose alteplase.

**Clinical Trial Registration:**

Clinicaltrials.gov identifier NCT01422616.


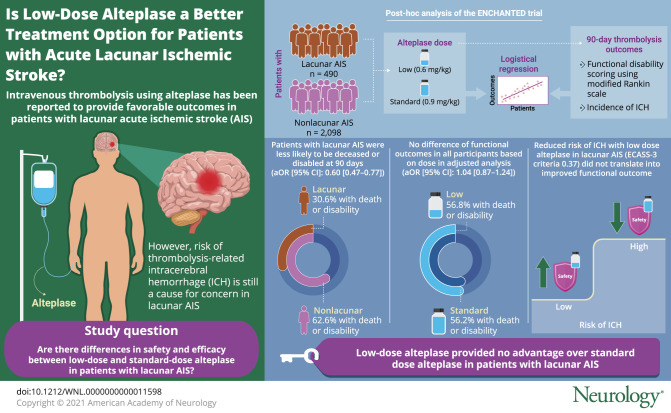


In routine clinical practice, patients with lacunar acute ischemic stroke (AIS) are eligible to receive IV thrombolysis, given comparable favorable outcomes to other common AIS pathologic subtypes.^1–3^ These results were confirmed in a recent subgroup analysis of the Efficacy and Safety of Magnetic Resonance Imaging–Based Thrombolysis in Wake-Up Stroke (WAKE-UP) trial, where the safety and efficacy of standard-dose IV alteplase were comparable between lacunar and nonlacunar subtypes defined on baseline MRI.^4^ Similar consistency of effect of IV alteplase between lacunar and nonlacunar AIS, defined by the Oxfordshire Community Stroke Project (OCSP) syndromic classification, was found in the third International Stroke Trial (IST-3).^5^ Despite this evidence, some clinical concern persists over whether the modest risk of thrombolysis-related intracerebral hemorrhage (ICH) could offset the modest benefits of IV thrombolysis for lacunar AIS, where the natural course is generally more benign compared to other AIS subtypes^6^ from there being no or small thrombotic lytic target on the presumption of a single penetrating artery occlusion.^7,8^

In the alteplase-dose arm of the Enhanced Control of Hypertension and Thrombolysis Stroke Study (ENCHANTED),^9^ a lower dose (0.6 mg/kg) of IV alteplase was shown to have a lower risk of ICH compared to standard dose (0.9 mg/kg) in thrombolysis-eligible patients with AIS. Whether it is the same for lacunar AIS is unclear. Herein, we report further analyses of the efficacy and safety of low- vs standard-dose IV alteplase in the ENCHANTED participants with lacunar (versus nonlacunar) AIS who were identified by the combination of clinical and adjudicated imaging findings.

## Methods

### Primary Research Question and Evidence Level

Is there are any differential efficacy and safety of low- vs standard-dose IV alteplase between participants with lacunar and nonlacunar AIS in the alteplase dose arm of the ENCHANTED trial? This study provides Class II evidence that for patients with lacunar AIS, low-dose alteplase has no additional benefit or safety over standard-dose alteplase.

### Design and Participants

ENCHANTED was an international, multicenter, 2 × 2 quasifactorial, prospective, randomized, open-label, blinded-endpoint trial that assessed the effectiveness of low-dose (0.6 mg/kg; 15% as bolus, 85% as infusion during 1 hour) vs standard-dose (0.9 mg/kg; 10% as bolus, 90% as infusion during 1 hour) IV alteplase, and more intensive vs guideline-recommended control of blood pressure (BP) in adult participants with AIS. The study design, participant characteristics, and main results of the alteplase-dose arm have been reported^9–11^ for 3,310 patients with AIS recruited from 111 centers in 13 countries. Key demographic and clinical characteristics were recorded at the time of enrollment, with clinical severity defined according to the NIH Stroke Scale (NIHSS) at baseline, 24 hours, and at day 7 (or on discharge from hospital if earlier). A final clinical diagnosis of AIS subtypes based upon the opinion of site investigations, generally according to the Trial of Org 10172 in Acute Stroke Treatment (TOAST) classification system,^12^ was made at day 7, postrandomization (or on discharge from hospital, if earlier).

### Standard Protocol Approvals, Registrations, and Participant Consents

The study protocol was approved by the appropriate ethics committee at each participating center and written informed consent was obtained from participants or an appropriate legal surrogate according to the Declaration of Helsinki. The ENCHANTED trial was registered at ClinicalTrials.gov (Unique identifier: NCT01422616).

### Imaging Analysis

Uncompressed digital images of all baseline and follow-up digital CT, MRI, and angiographic images were uploaded into the study brain imaging database in Digital Imaging and Communications in Medicine (DICOM) format identified only by the participant's unique study identification number. Images were analyzed centrally for any ICH by a trial adjudication panel, blind to clinical data, treatment, date, and sequence of scan. Assessors graded any identified symptomatic ICH (sICH) using a range of standard definitions from the Safe Implementation of Thrombolysis in Stroke–Monitoring Study (SITS-MOST), National Institute of Neurologic Disease and Stroke (NINDS), the European–Australian Cooperative Acute Stroke Study II (ECASS), ECASS III, and IST-3 (additional Methods I, doi.org/10.5061/dryad.t1g1jwt0s).

The ENCHANTED Imaging Analysis Project was established in August 2016, with the aim of defining the presence, extent, and severity of, and swelling from, acute ischemic changes (including arterial territory, border zone, small subcortical and brainstem/cerebellar infarcts), coexisting old vascular lesions and their subtypes, white matter lesions, and brain volume loss on all collected images by an imaging analysis team of trained individuals, blind to all clinical data, using an electronic scoring system modified from IST-3.^13^ All observed infarct lesions on baseline (prerandomization) CT or MRI were coded according to the IST-3 criteria for infarct site and size. Separately and subsequent to primary scan reads, a neuroradiologist (Z.Z.) and neurosurgeon (X.C.) sought the ischemic lesion on 24-hour follow-up images while viewing the baseline images for those with no infarct lesion identified at baseline. They also assessed large vessel occlusion (LVO) on baseline CT angiography (CTA) or magnetic resonance angiography (MRA) according to a modified Thrombolysis in Cerebral Infarction (TICI) score for an abnormal artery in IST-3.^14^ All the imaging data were cross-checked (Z.Z.) and a final rating made before unmasking the clinical data and randomization code for analyses.

### Definitions of Lacunar and Nonlacunar AIS

**Figure 1:**
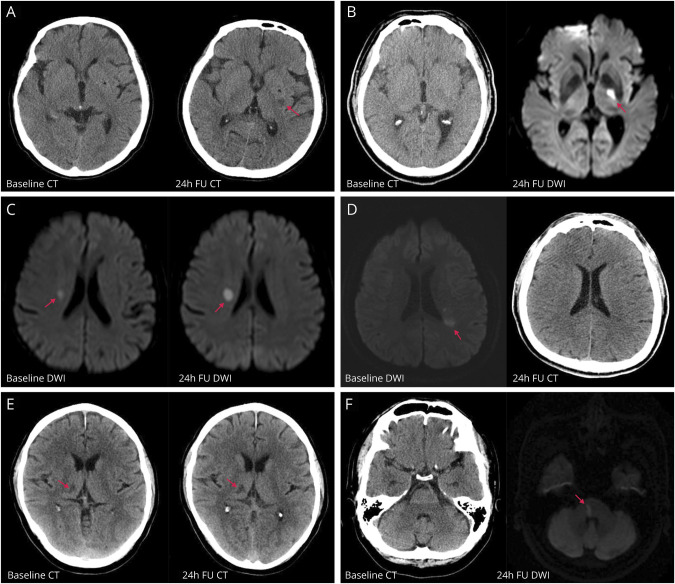
Examples of Lacunar Ischemic Stroke at Different Locations From ENCHANTED Lacunar stroke at (A) left lentiform (red arrow) identified on 24-hour follow-up CT; (B) left internal capsule (red arrow) identified on 24-hour follow-up MRI; (C) right centrum semiovale (red arrow) identified on baseline and 24-hour follow-up MRI; (D) left internal border zone (red arrow) identified on baseline MRI; (E) right thalamus (red arrow) identified on baseline and 24-hour follow-up CT; and (F) brainstem (red arrow) identified on 24-hour follow-up MRI. DWI = diffusion-weighted imaging; ENCHANTED = Enhanced Control of Hypertension and Thrombolysis Stroke Study.

Different levels of confidence (definite/probable/possible) were used around the definitions of lacunar and nonlacunar AIS based on adjudicated imaging findings, clinical severity, and clinical diagnosis (additional Methods II, doi.org/10.5061/dryad.t1g1jwt0s). In brief, definite lacunar AIS was defined when all 4 criteria were met: (1) the presence of acute infarct lesion (maximum diameter ≤20 mm) in the territory of penetrating arteries, with a rounded, ovoid, or tubular shape on axial CT or diffusion-weighted imaging/apparent diffusion coefficient map (typical examples are shown in figure 1)^15^; (2) no LVO adjudicated centrally (on CTA/MRA) or reported by site investigators (on CTA/MRA/digital subtraction angiography); (3) the final diagnosis was reported as “small vessel or perforating vessel ‘lacunar’ disease” according to the TOAST criteria that involved any of the standard clinical lacunar syndromes with the lack of large vessel atheroma or cerebral cortical dysfunction; and (4) infarct side on images is consistent with that reported by site investigators. Definite nonlacunar AIS was defined as having acute infarct lesion with maximum diameter >20 mm or LVO on angiography. Participants were classified as nonlacunar if they had lacunar and nonlacunar infarcts.

Given that the clinical diagnosis of lacunar syndrome plus baseline NIHSS score <7 had a high specificity to predict imaging-confirmed lacunar stroke in IST-3,^16^ probable lacunar and nonlacunar AIS were discriminated mainly by baseline NIHSS scores and final diagnosis in the situation that there was no acute infarct lesion identified on images or the images were not collected from the sites. For those with conflicting clinical and adjudicated imaging information that compromised the confidence of discrimination, we classified as possible lacunar or nonlacunar AIS according to the clinical diagnosis and LVO status.

### Outcomes

The primary outcome of these analyses was the composite endpoint of disability or death (modified Rankin Scale [mRS] scores 2–6) at 90 days postrandomization. Secondary efficacy outcomes included major disability or death (mRS 3–6), death (mRS 6), and ordinal shift of the full range of mRS scores at 90 days. Secondary safety outcomes were sICH defined according to several criteria from other studies, fatal ICH within 7 days, ICH identified by central adjudicators, and any ICH adjudicated centrally or reported by site investigators. Other clinical outcomes included early neurologic deterioration (END) (≥4-point increase in NIHSS scores) or death within 24 hours or 7 days.

### Statistical Analysis

Continuous or categorical variables at baseline were presented as mean (SD), median (interquartile range), or number (percentage). Baseline differences between participants with lacunar and nonlacunar AIS were evaluated using analysis of variance, χ^2^ test, or Wilcoxon signed-rank test, as appropriate. Associations of lacunar AIS with 90-day function, safety, and other secondary outcomes were estimated in logistic regression models with adjustment for randomized treatment and key prognostic covariates (age, sex, ethnicity, baseline NIHSS score, time from symptom onset to randomization, premorbid function [mRS score 0 or 1], prior use of antithrombotic agents, history of diabetes or cardiovascular disease, and assigned to intensive blood pressure–lowering group). The treatment effect of low- vs standard-dose alteplase was determined in logistic regression models and the heterogeneity of alteplase dose effect across participants with lacunar and nonlacunar AIS was estimated by adding an interaction term to statistical models. Proportional odds regression models were used to analyze ordinal mRS scores. The primary analyses pertain to participants with definite/probable lacunar and nonlacunar AIS after excluding those with a possible diagnostic classification. Sensitivity analyses of the treatment effects of low- vs standard-dose alteplase were performed in participants with definite lacunar/nonlacunar AIS and in all participants with possible lacunar/nonlacunar AIS. We also performed an exploratory analysis of the treatment effects in a subset of lacunar AIS identified at baseline (infarct size ≤15 mm and no adjudicated LVO). Data were reported as odds ratios (ORs) and 95% confidence intervals (CIs) and a 2-sided *p* < 0.05 was considered statistically significant. All analyses were performed using SAS version 7.1 and Stata version 12.0.

### Data Availability

Additional methods (I and II) and data (supplementary tables 1–3) are available from Dryad (doi.org/10.5061/dryad.t1g1jwt0s). Individual de-identified participant data used in this analysis will be shared by request from any qualified investigator via the Research Office of The George Institute for Global Health.

## Results

### Baseline Characteristics

**Figure 2:**
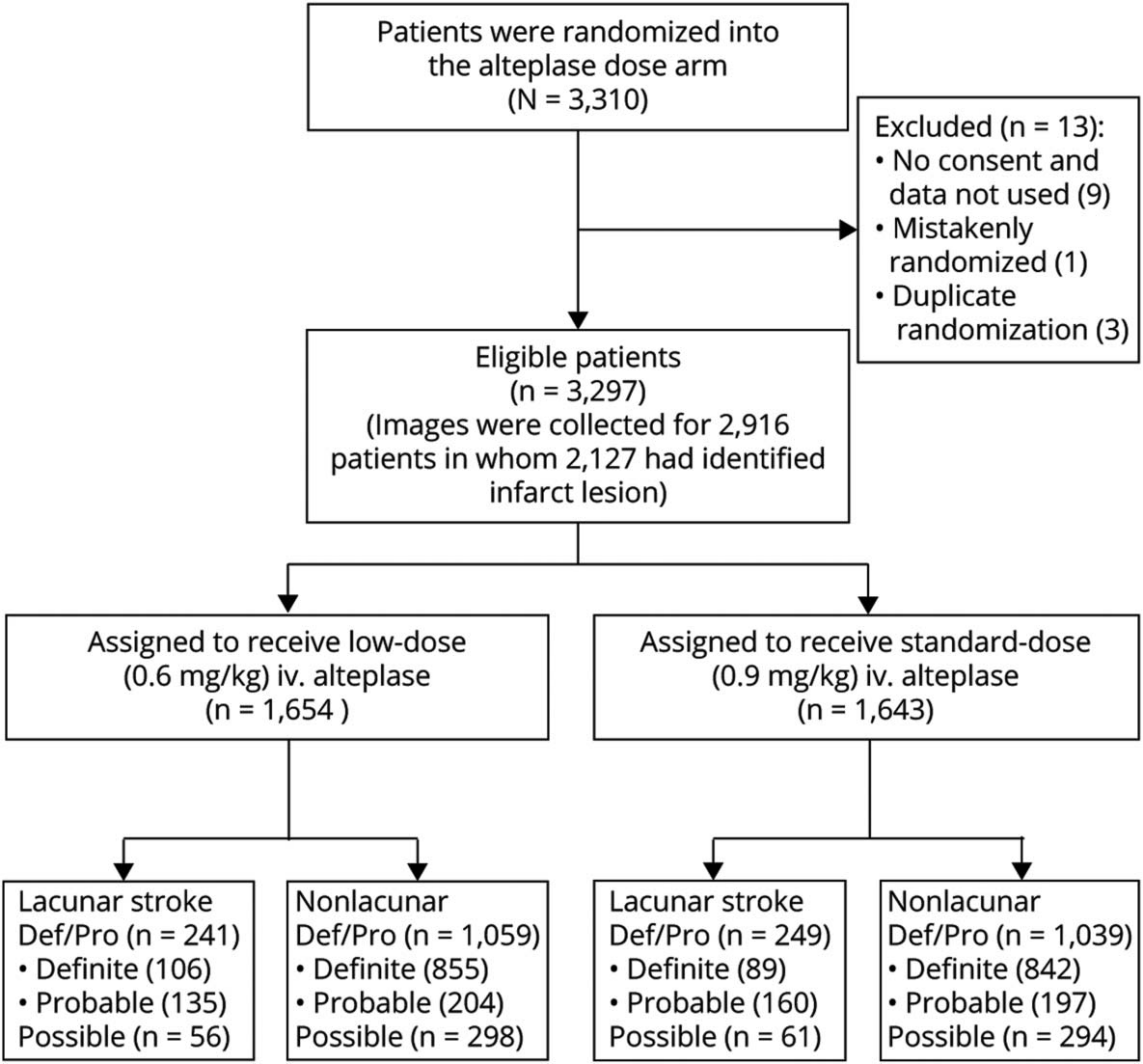
Flowchart of Participants Included in Analyses Def/Pro = definite or probable.

Among 3,297 AIS participants in the ENCHANTED alteplase dose arm, 2,588 (78.5%) were classifiable (definite lacunar, n = 195; probable lacunar, n = 295; definite nonlacunar, n = 1,697; and probable nonlacunar, n = 401 AIS) for inclusion in the primary analysis (figure 2). Compared to the 709 excluded participants, they were more likely to be older, have higher baseline NIHSS scores, be Asian, have a history of cardiovascular disease, and have a final diagnosis of LVO, but they also had shorter time interval from symptom onset to randomization (supplementary table 1, doi.org/10.5061/dryad.t1g1jwt0s). Table 1 shows that all the baseline clinical characteristics were significantly different between definite/probable lacunar and nonlacunar AIS except for sex, history of diabetes, and prior use of statin/other lipid-lowering agents. Participants with lacunar (versus nonlacunar) AIS were younger and had milder neurologic impairment, higher baseline BP, and a lower proportion with conventional cardiovascular risk factors except smoking. In keeping with the lacunar pattern of stroke, few participants had multiple lesions in both anterior and posterior circulation, but they were more likely to have a lesion only in the posterior circulation. They were also less likely to have brain atrophy or a hyperdense vessel sign on CT or hyperintense arteries on MRI.

**Table 1:**
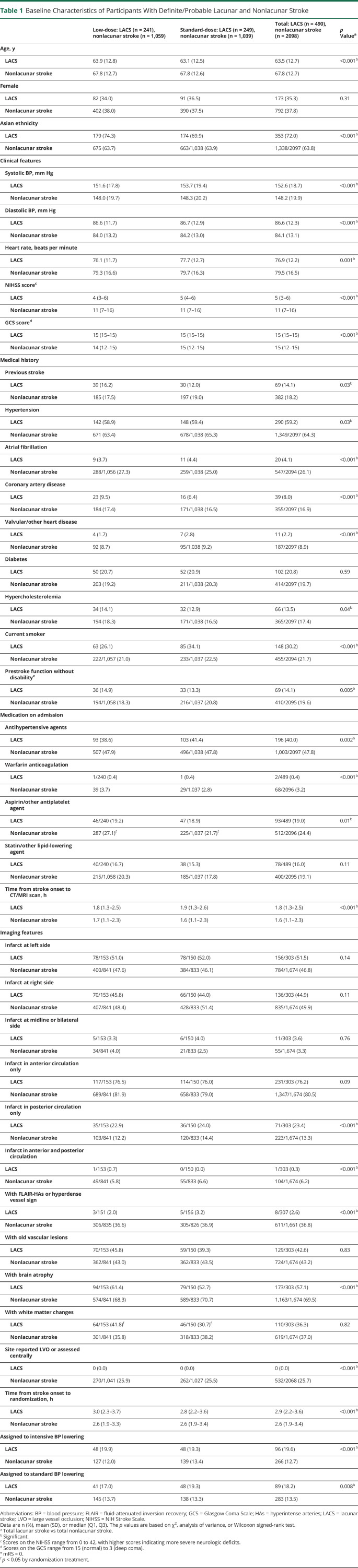
Baseline Characteristics of Participants With Definite/Probable Lacunar and Nonlacunar Stroke

### Lacunar AIS and Outcomes

Compared to participants with definite/probable nonlacunar AIS, those with definite/probable lacunar AIS had better 90-day functional outcomes, whether defined by the outcome of mRS scores 2–6 (unadjusted OR 0.26, 95% CI 0.21–0.33), mRS scores 3–6 (0.20, 0.15–0.26), ordinal shift in the full range of scores (0.27, 0.23–0.33), or death alone (0.04, 0.01–0.12) (table 2). They were also less likely to have ICH and END or death after IV thrombolysis. The findings persisted with adjustment of baseline covariables and randomized alteplase dose.

**Table 2:**
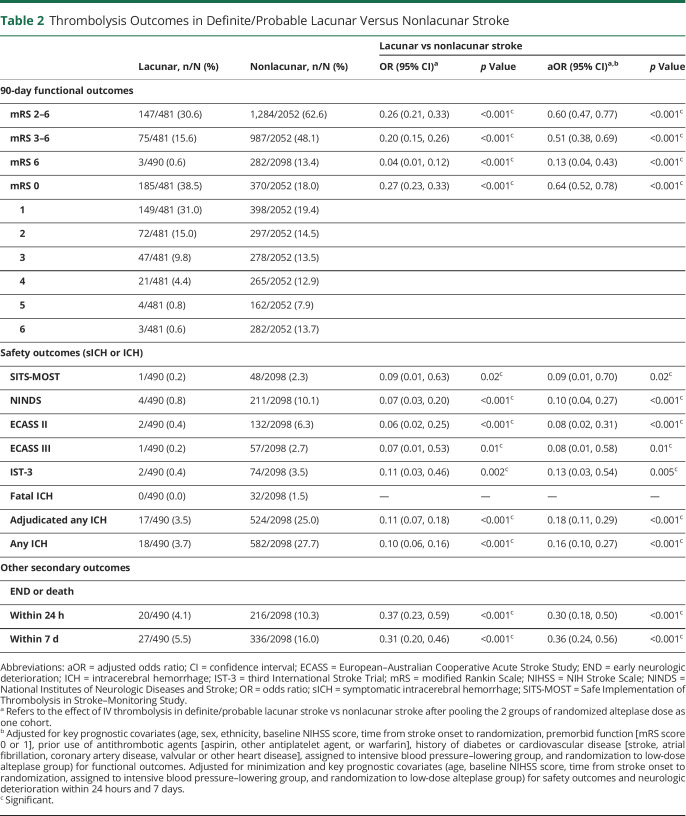
Thrombolysis Outcomes in Definite/Probable Lacunar Versus Nonlacunar Stroke

### Lacunar AIS and Alteplase Dose

**Figure 3:**
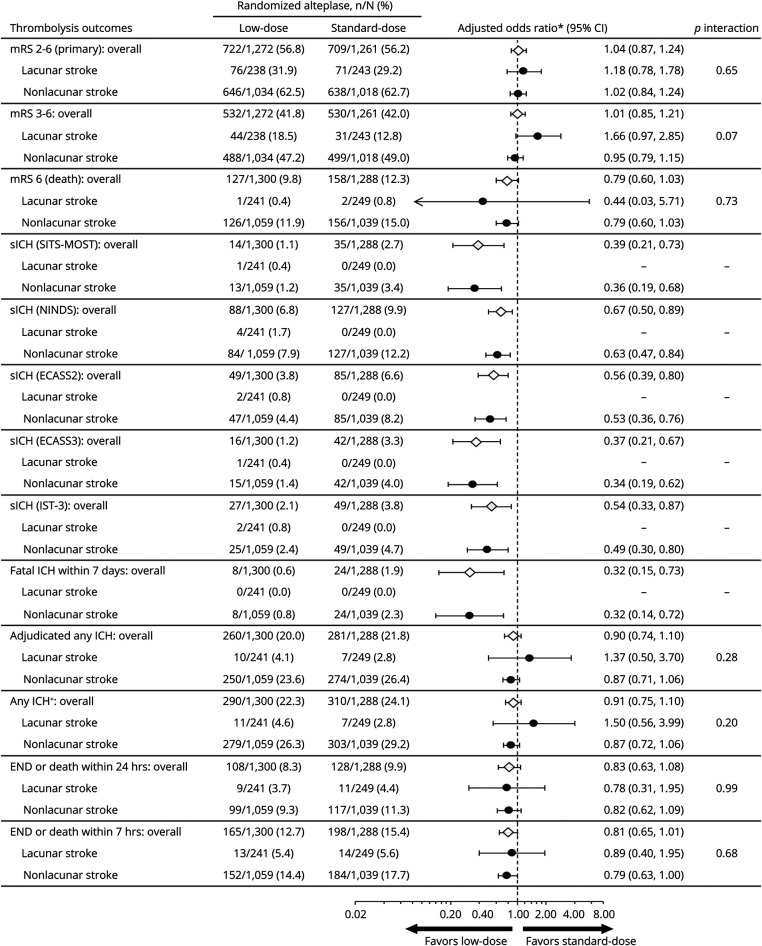
Thrombolysis Outcomes in Participants With Definite/Probable Lacunar and Nonlacunar Stroke by Randomized Treatment *Adjusted for key prognostic covariates (age, sex, ethnicity, baseline NIH Stroke Scale [NIHSS] score, time from stroke onset to randomization, premorbid function [modified Rankin Scale (mRS) scores 0 or 1], prior use of antithrombotic agents [aspirin, other antiplatelet agent, or warfarin], history of diabetes or cardiovascular disease [stroke, atrial fibrillation, coronary artery disease, valvular or other heart disease], assigned to intensive blood pressure–lowering group) for functional outcomes. Adjusted for minimization and key prognostic covariates (age, baseline NIHSS score, time from stroke onset to randomization, and assigned to intensive blood pressure–lowering group) for safety outcomes and neurologic deterioration within 24 hours or 7 days.†Site reported or adjudicated centrally. CI = confidence interval; ECASS = European–Australian Cooperative Acute Stroke Study; END = early neurologic deterioration; ICH = intracerebral hemorrhage; IST-3 = third International Stroke Trial; NINDS = National Institutes of Neurologic Diseases and Stroke; sICH = symptomatic intracerebral hemorrhage; SITS-MOST = Safe Implementation of Thrombolysis in Stroke–Monitoring Study.

**Figure 4:**
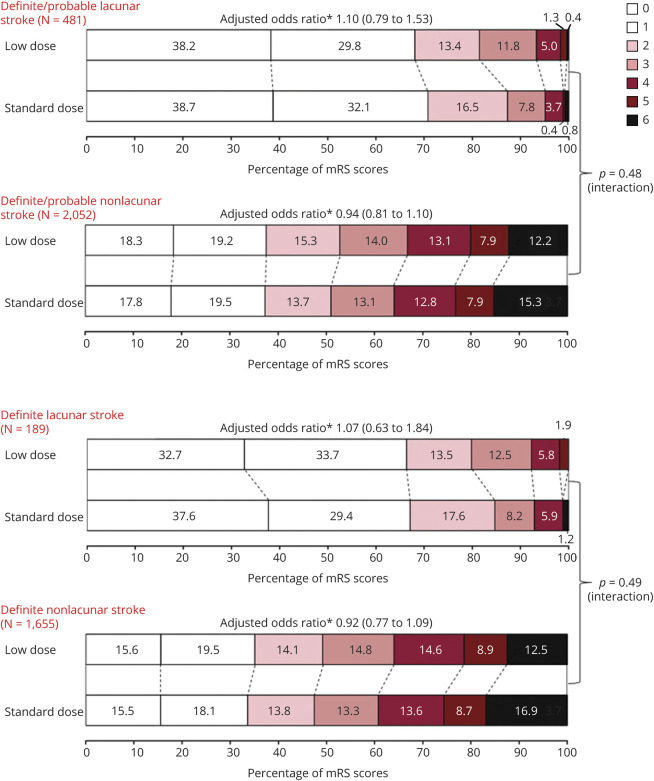
Randomized Treatment Effects on the Ordinal Modified Rankin Scale (mRS) Score by Lacunar and Nonlacunar Stroke *Adjusted for key prognostic covariates (age, sex, ethnicity, baseline NIH Stroke Scale [NIHSS] score, time from stroke onset to randomization, premorbid function [mRS scores 0 or 1], prior use of antithrombotic agents [aspirin, other antiplatelet agent, or warfarin], history of diabetes or cardiovascular disease [stroke, atrial fibrillation, coronary artery disease, valvular or other heart disease], assigned to intensive blood pressure–lowering group).

**Figure 5:**
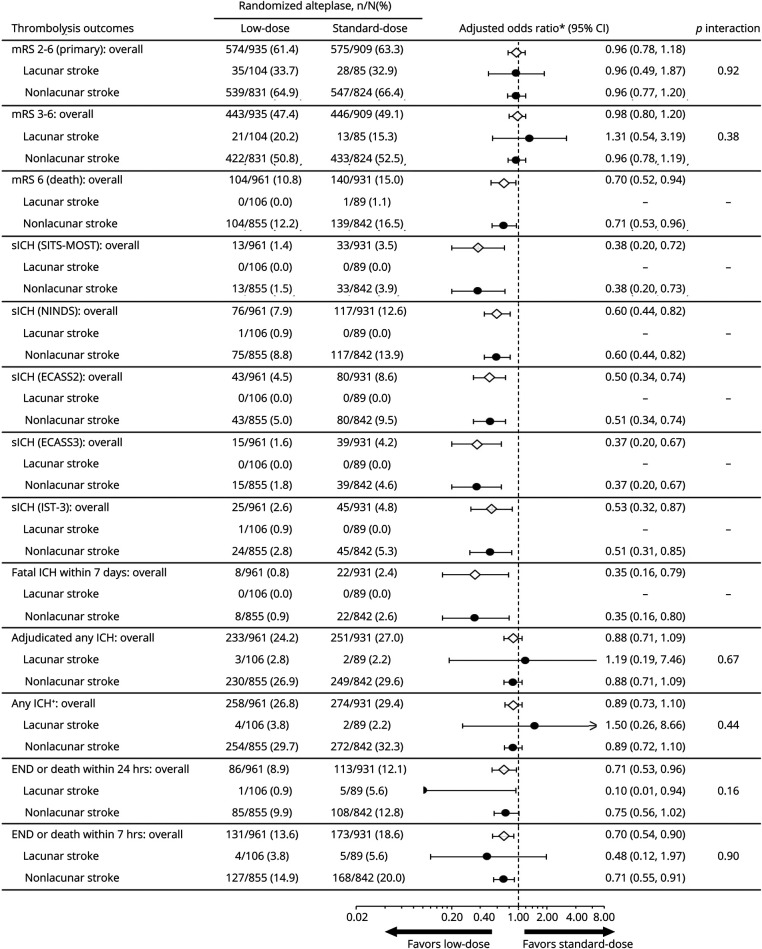
Thrombolysis Outcomes in Participants With Definite Lacunar and Nonlacunar Stroke by Randomized Treatment *Adjusted for key prognostic covariates (age, sex, ethnicity, baseline NIH Stroke Scale [NIHSS] score, time from stroke onset to randomization, premorbid function [modified Rankin Scale (mRS) scores 0 or 1], prior use of antithrombotic agents [aspirin, other antiplatelet agent, or warfarin], history of diabetes or cardiovascular disease [stroke, atrial fibrillation, coronary artery disease, valvular or other heart disease], assigned to intensive blood pressure–lowering group) for functional outcomes. Adjusted for minimization and key prognostic covariates (age, baseline NIHSS score, time from stroke onset to randomization, and assigned to intensive blood pressure–lowering group) for safety outcomes and neurologic deterioration within 24 hours or 7 days. †Site reported or adjudicated centrally. CI = confidence interval; ECASS = European–Australian Cooperative Acute Stroke Study; END = early neurologic deterioration; ICH = intracerebral hemorrhage; IST-3 = third International Stroke Trial; NINDS = National Institutes of Neurologic Diseases and Stroke; sICH = symptomatic intracerebral hemorrhage; SITS-MOST = Safe Implementation of Thrombolysis in Stroke–Monitoring Study.

The overall treatment effects of low- vs standard-dose alteplase on function, safety, and other outcomes in these 2,588 participants were comparable to the main results of the ENCHANTED trial, that low-dose vs standard-dose alteplase reduced the risk of sICH (SITS-MOST criteria, adjusted OR 0.39, 95% CI 0.21–0.73; NINDS criteria, 0.67, 0.50–0.89; ECASS II criteria, 0.56, 0.39–0.80; ECASS III criteria, 0.37, 0.21–0.67; IST-3 criteria, 0.54, 0.33–0.87) but with no difference in effect on functional outcomes (mRS 2–6, adjusted OR 1.04, 95% CI 0.87–1.24; mRS 3–6, 1.01, 0.85–1.21). There was no heterogeneity of treatment effects on all outcomes for definite/probable lacunar vs nonlacunar AIS after adjustment for baseline covariables (all *p*_interaction_ ≥0.07) (figures 3 and 4). Similar results were seen in the sensitivity analyses for definite lacunar and nonlacunar AIS (all *p*_interaction_ ≥0.16) (figures 4 and 5) and definite/probable/possible lacunar and nonlacunar AIS (all *p*_interaction_ ≥0.12) (data available on request).

Specifically, in the definite subgroup of lacunar AIS, there were no significant differences on the primary efficacy outcome (mRS 2–6) (33.7% vs 32.9%, adjusted OR 0.96, 95% CI 0.49–1.87) or major disability or death (mRS 3–6) (20.2% vs 15.3%, adjusted OR 1.31, 95% CI 0.54–3.19) between low-dose and standard-dose alteplase groups (figure 5). There was one case of sICH (0.9%) meeting NINDS and IST-3 criteria in participants with definite lacunar AIS treated by low-dose alteplase, but no case of sICH was observed after use of standard-dose alteplase. In participants with definite lacunar AIS who received low-dose alteplase, 3 (2.8%) had adjudicated ICH and 1 more had ICH reported by a site investigator, while any ICH occurred in 2 (2.2%) participants with definite lacunar AIS assigned to the standard-dose group. In a smaller subset of definite lacunar AIS identified at baseline with size <15 mm and no adjudicated LVO, 4 of the 9 participants (44.4%) in the low-dose group and 2 of the 7 participants (28.6%) in the standard-dose group had mRS 2–6 at 90 days postrandomization, and no ICH occurred in either treatment group (supplementary table 2, doi.org/10.5061/dryad.t1g1jwt0s).

## Discussion

In these post hoc analyzes of the ENCHANTED trial, we did not identify any benefit, nor any harm, from the use of low-dose alteplase vs standard-dose alteplase to treat patients with lacunar AIS compared to those with other subtypes of AIS. As well as having a range of significantly different characteristics, the 90-day outcomes were better for those with lacunar than nonlacunar AIS, which provided some internal consistency for the classifications used in our study. However, given the low event rate of sICH, with fewer than 5 events in the primary analysis for definite or probable lacunar AIS, we are limited in the conclusions that can be drawn as to whether a lower dose of IV alteplase should be preferred because of the good prognosis for lacunar AIS.

Our results on thrombolysis outcomes for lacunar AIS are consistent with prior observational studies.^2,17–21^ However, the net benefit of thrombolysis for lacunar AIS is still debated, mainly because the evidence is drawn from subgroup analyzes of trials, such as WAKE-UP^4^ and IST-3,^5^ where there is low statistical power. In addition, accurate identification of lacunar AIS is challenging, especially in the absence of an acute lesion on the initial CT, and even MRI (in nearly one third of patients with nondisabling stroke).^22^ The pragmatic approach of applying a lacunar syndrome classification system in studies has moderate diagnostic sensitivity and specificity,^15^ which may potentially mix patients with nonlacunar AIS with the target population of lacunar AIS, and nondifferentially bias results towards IV thrombolysis.

We were unable to confirm in ENCHANTED participants any benefit of low-dose over standard-dose alteplase in lacunar AIS. The fact that there were few cases of sICH in the low-dose alteplase group, and no sICH in the standard-dose group, highlights the potential for chance and imprecise estimates of treatment effects when there are few events. Even with current imaging techniques and clinical criteria, it is difficult to discriminate lacunar AIS due to occlusion of a deep penetrating arteriole presumed caused by progressive lipohyalinosis from thrombosis related to atherosclerosis or embolus. Platelet activation triggered by disintegration of the endothelium from intrinsic cerebral small vessel disease (CSVD) may also be relevant in this type of AIS.^8^ It is possible, therefore, that IV thrombolysis may have a differential effect dependent on the cause of lacunar stroke, being more effective when there is underlying thromboembolism. In lacunar AIS, we noted a significant imbalance in the frequency of background white matter lesions between the low-dose and standard-dose alteplase groups (41.8% vs 30.7%), which could partly account for more ICH in the former (supplementary table 3, doi.org/10.5061/dryad.t1g1jwt0s).^23^ Again, however, due to the few sICH events in patients with lacunar AIS, we cannot confirm whether the increase in sICH by low-dose alteplase was confounded by CSVD.

Some strengths of our study include the large, prospective, multicenter cohort of patients with AIS who had systematic, complete, and high-quality data collected prospectively, where we were able to adjust for multiple covariables in statistical models. Furthermore, the imaging assessment was completed blind to clinical features and other data, using a rigorously defined approach developed for the IST-3 study. However, we acknowledge limitations that include insufficient statistical power and inevitable selection bias from the data being derived from a clinical trial where a large number of participants were from Asia and had mild to moderate stroke. Moreover, given the pragmatic nature of ENCHANTED, few participants had a baseline brain MRI, and the identification of lacunar AIS required analysis of follow-up images with comparison to those obtained at baseline. Whereas this approach may have altered the imaging appearances of acute ischemic lesions after use of IV thrombolysis^24^ and limited the identification of all true lacunar AIS, our results are comparable with previous work showing that nearly one-third of patients with nondisabling AIS lack an infarct lesion on acute MRI (median 4 days poststroke).^22^ In the ENCHANTED alteplase arm, 27.1% (789/2,916) of participants had no infarct lesion on either the baseline or 24-hour follow-up images. Thus, we had to use a combination of clinical and adjudicated imaging data to classify as many cases as possible into lacunar and nonlacunar AIS, which likely closely represents that used in routine practice. Relatively small samples in lacunar AIS compromised the power of a reliable assessment of any interaction, especially for sICH. Moreover, regarding the outcomes of major disability or death, a *p*_interaction_ of 0.07 might have been due to chance rather than true differential treatment effects of low- vs standard-dose alteplase across definite/probable lacunar and nonlacunar AIS. Future research in systematic reviews and clinical registries may be required to confirm or refute these findings.

We found no clear evidence that low-dose IV alteplase was any better or safer than standard-dose alteplase in the ENCHANTED participants who had lacunar AIS. According to standard eligibility criteria, patients with lacunar AIS should receive standard dose IV alteplase as with other AIS subtypes.

## Glossary

AIS: acute ischemic stroke
BP: blood pressure
CI: confidence interval
CSVD: cerebral small vessel disease
CTA: CT angiography
DICOM: Digital Imaging and Communications in Medicine
ECASS: European–Australian Cooperative Acute Stroke Study
ENCHANTED: Enhanced Control of Hypertension and Thrombolysis Stroke Study
END: early neurologic deterioration
ICH: intracerebral hemorrhage
IST-3: the third International Stroke Trial
LVO: large vessel occlusion
MRA: magnetic resonance angiography
mRS: modified Rankin Scale
NIHSS: NIH Stroke Scale
NINDS: National Institutes of Neurological Diseases and Stroke
OCSP: Oxfordshire Community Stroke Project
OR: odds ratio
sICH: symptomatic intracerebral hemorrhage
SITS-MOST: the Safe Implementation of Thrombolysis in Stroke–Monitoring Study
TICI: Thrombolysis in Cerebral Infarction
TOAST: Trial of Org 10172 in Acute Stroke Treatment classification
WAKE-UP: Efficacy and Safety of MRI-based Thrombolysis in Wake-Up Stroke trial

## References

[1] Hsia AW, Sachdev HS, Tomlinson J, et al. Efficacy of IV tissue plasminogen activator in acute stroke: does stroke subtype really matter? Neurology 2003;61:71–75. 12847159 10.1212/01.wnl.0000071228.56362.36

[2] Fuentes B, Martínez-Sánchez P, Alonso de Leciñana M, et al. Efficacy of intravenous thrombolysis according to stroke subtypes: the Madrid Stroke Network data. Eur J Neurol 2012;19:1568–1574. 22742869 10.1111/j.1468-1331.2012.03790.x

[3] Mustanoja S, Meretoja A, Putaala J, et al. Outcome by stroke etiology in patients receiving thrombolytic treatment: descriptive subtype analysis. Stroke 2011;42:102–106. 21106955 10.1161/STROKEAHA.110.597534

[4] Barow E, Boutitie F, Cheng B, et al. Functional outcome of intravenous thrombolysis in patients with lacunar infarcts in the WAKE-UP trial. JAMA Neurol 2019;76:641–649. 30907934 10.1001/jamaneurol.2019.0351 PMC6563546

[5] Lindley RI, Wardlaw JM, Whiteley WN, et al. Alteplase for acute ischemic stroke: outcomes by clinically important subgroups in the third International Stroke Trial. Stroke 2015;46:746–756. 25613308 10.1161/STROKEAHA.114.006573

[6] Bamford J, Sandercock P, Jones L, et al. The natural history of lacunar infarction: the Oxfordshire Community Stroke Project. Stroke 1987;18:545–551. 3590244 10.1161/01.str.18.3.545

[7] Norrving B. Lacunar infarcts: no black holes in the brain are benign. Pract Neurol 2008;8:222–228. 18644908 10.1136/jnnp.2008.153601

[8] Regenhardt RW, Das AS, Lo EH, Caplan LR. Advances in understanding the pathophysiology of lacunar stroke: a review. JAMA Neurol 2018;75:1273–1281. 30167649 10.1001/jamaneurol.2018.1073 PMC7426021

[9] Anderson CS, Robinson T, Lindley RI, et al. Low-dose versus standard-dose intravenous alteplase in acute ischemic stroke. N Engl J Med 2016;374:2313–2323. 27161018 10.1056/NEJMoa1515510

[10] Huang Y, Sharma VK, Robinson T, et al. Rationale, design, and progress of the Enhanced Control of Hypertension and Thrombolysis Stroke Study (ENCHANTED) trial: an international multicenter 2 × 2 quasi-factorial randomized controlled trial of low- vs. standard-dose rt-PA and early intensive vs. guideline-recommended blood pressure lowering in patients with acute ischaemic stroke eligible for thrombolysis treatment. Int J Stroke 2015;10:778–788. 25832995 10.1111/ijs.12486

[11] Anderson CS, Woodward M, Arima H, et al. Statistical analysis plan for evaluating low- vs. standard-dose alteplase in the Enhanced Control of Hypertension and Thrombolysis Stroke Study (ENCHANTED). Int J Stroke 2015;10:1313–1315. 26283139 10.1111/ijs.12602 PMC5324659

[12] Adams HP Jr, Bendixen BH, Kappelle LJ, et al. Classification of subtype of acute ischemic stroke: definitions for use in a multicenter clinical trial: TOAST: Trial of Org 10172 in Acute Stroke Treatment. Stroke 1993;24:35–41. 7678184 10.1161/01.str.24.1.35

[13] Wardlaw JM, Sandercock P, Cohen G, et al. Association between brain imaging signs, early and late outcomes, and response to intravenous alteplase after acute ischaemic stroke in the third International Stroke Trial (IST-3): secondary analysis of a randomised controlled trial. Lancet Neurol 2015;14:485–496. 25819484 10.1016/S1474-4422(15)00012-5 PMC4513190

[14] Mair G, von Kummer R, Adami A, et al. Arterial obstruction on computed tomographic or magnetic resonance angiography and response to intravenous thrombolytics in ischemic stroke. Stroke 2017; 48:353–360. 28008093 10.1161/STROKEAHA.116.015164 PMC5266422

[15] Wardlaw JM, Smith EE, Biessels G, et al. Neuroimaging standards for research into small vessel disease and its contribution to ageing and neurodegeneration. Lancet Neurol 2013;12:822–838. 23867200 10.1016/S1474-4422(13)70124-8 PMC3714437

[16] Arba F, Mair G, Phillips S, et al. Improving clinical detection of acute lacunar stroke: analysis from the IST-3. Stroke 2020;51:1411–1418. 32268853 10.1161/STROKEAHA.119.028402 PMC7185055

[17] Matusevicius M, Paciaroni M, Caso V, et al. Outcome after intravenous thrombolysis in patients with acute lacunar stroke: an observational study based on SITS international registry and a meta-analysis. Int J Stroke 2019;14:878–886. 30935349 10.1177/1747493019840947

[18] Zivanovic Z, Gubi M, Vlahovic D, et al. Patients with acute lacunar infarction have benefit from intravenous thrombolysis. J Stroke Cerebrovasc Dis 2019;28:435–440. 30409747 10.1016/j.jstrokecerebrovasdis.2018.10.020

[19] Eggers CCJ, Bocksrucker C, Seyfang L, et al. The efficacy of thrombolysis in lacunar stroke: evidence from the Austrian Stroke Unit Registry. Eur J Neurol 2017;24:780–787. 28449276 10.1111/ene.13288

[20] Shobha N, Fang J, Hill MD. Do lacunar strokes benefit from thrombolysis? Evidence from the registry of the Canadian Stroke Network. Int J Stroke 2013;8(suppl A100):45–49. 23231478 10.1111/j.1747-4949.2012.00932.x

[21] Fluri F, Hatz F, Rutgers MP, et al. Intravenous thrombolysis in patients with stroke attributable to small artery occlusion. Eur J Neurol 2010;17:1054–1060. 20136649 10.1111/j.1468-1331.2010.02961.x

[22] Makin SD, Doubal FN, Dennis MS, et al. Clinically confirmed stroke with negative diffusion-weighted imaging magnetic resonance imaging: longitudinal study of clinical outcomes, stroke recurrence, and systematic review. Stroke 2015;46:3142–3148. 26419965 10.1161/STROKEAHA.115.010665 PMC4617292

[23] Pantoni L, Fierini F, Poggesi A. Thrombolysis in acute stroke patients with cerebral small vessel disease. Cerebrovasc Dis 2014;37:5–13. 24355873 10.1159/000356796

[24] Nagaraja N, Forder JR, Warach S, et al. Reversible diffusion-weighted imaging lesions in acute ischemic stroke: a systematic review. Neurology 2020;94:571–587. 32132175 10.1212/WNL.0000000000009173

